# Antitoxin in Distemper

**Published:** 1902-02

**Authors:** H. A. Stevenson

**Affiliations:** London, Ontario


### ANTITOXIN IN DISTEMPER.

By H. A. Stevenson, M.D.,
LONDON, ONTARIO.

The antitoxin treatment of certain diseases has a certain class of enemies who are opposed to this manner of treatment. I have always been an advocate of this method of treatment, and so I wish to relate one interesting case that has come under my notice lately and add one testimony in its favor.

I had a young Irish setter bitch which, when pregnant about four weeks, contracted a severe attack of distemper. I tried all the remedies known to give relief, but they failed to do her any good, and she gradually grew worse, and I thought that I was going to lose her. She had running at the eyes, fever, a thick, creamy discharge from both nostrils, and lost flesh very rapidly—became almost a skeleton. I obtained some of Mulford's distemper antitoxin, and gave her one moderate-sized dose as a last resort. I never saw in any patient such a change, and that very suddenly. In twenty-four hours she began to improve; in forty-eight hours the discharge from the nose stopped. She aborted at nearly full term, but we killed the pups, as they were too much trouble to her; she rapidly got well, and in a week she was able to go for a short run with the horse. I believe the antitoxin certainly worked well in this case; but this is only one case, and it may fail in others. I certainly will use it in all cases in future that I come across.

